# Clinical and genetic analysis of distal renal tubular acidosis in three Chinese children

**DOI:** 10.1080/0886022X.2018.1487858

**Published:** 2018-09-19

**Authors:** Jiaojiao Liu, Qian Shen, Guomin Li, Yihui Zhai, Xiaoyan Fang, Hong Xu

**Affiliations:** Department of Nephrology, Children’s Hospital of Fudan University, Shanghai, China

**Keywords:** ATP6V0A4, ATP6V1B1, SLC4A1, distal renal tubular acidosis, growth, recombinant human growth hormone

## Abstract

**Objective**: Primary distal renal tubular acidosis (dRTA) is a rare genetic disease characterized by distal tubular dysfunction leading to metabolic acidosis and alkaline urine. Growth retardation is a major concern in these children. The disease is caused by defects in at least three genes (SLC4A1, ATP6V0A4, and ATP6V1B1) involved in urinary distal acidification. Several series of dRTA patients from different ethnic backgrounds have been genetically studied, but genetic studies regarding Chinese population is rare. Our aim was to investigate the clinical features and genetic basis of primary dRTA in Chinese children.

**Methods:** Three unrelated patients with dRTA participated in our study. Next-generation sequencing was performed, and the findings were validated using the Sanger sequencing method.

**Results:** All patients exhibited hyperchloraemic metabolic acidosis, abnormally high urine pH, hypokalemia, and nephrocalcinosis. Growth retardation was observed in all patients. During the follow-up (range 1–4 years), alkali replacement therapy corrected the systemic metabolic acidosis, and two patients demonstrated normal growth. rhGH therapy was administered to patient-3 at the age of 6 years, and his growth rate was significantly improved (growth velocity 9.6 cm/yr). In total, 5 mutations were identified in our cohort of three patients, and four mutations were novel.

**Conclusions:** We report the clinical and molecular characteristics of dRTA patients from China. The four novel mutations detected in our study extend the spectrum of gene mutations associated with primary dRTA. Furthermore, our study confirms the effect of early treatment in improving growth for dRTA patient and provides insight into the effects of rhGH on dRTA patients who were diagnosed late and exhibiting a persistent growth delay despite appropriate therapy.

## Introduction

Renal tubular acidosis (RTA) is a clinical syndrome resulting from renal tubular failure of acid secretion and/or bicarbonate reabsorption that leads to metabolic acidosis with a normal anion gap (hyperchloraemic). RTA can represent a primary or secondary defect. Primary defects are common in children due to gene mutations or the idiopathic nature of the syndrome, whereas secondary defects are more common in adults. RTA is classified into the following four types based on the clinical features: distal renal tubular acidosis (type I), proximal renal tubular acidosis (type II), combined proximal and distal renal tubular acidosis (type III), and hyperkalaemic renal tubular acidosis (type IV) [1]. Primary dRTA is an inherited disorder caused by mutations in at least three genes (SLC4A1, ATP6V0A4, and ATP6V1B1) [2]. Both autosomal-dominant and autosomal-recessive forms of dRTA have been described.

Several series of dRTA patients from different ethnic backgrounds have been genetically studied [3–13]. To date, dRTA cases caused by gene mutations in the Chinese population have rarely been reported [14,15]. In sporadic cases, clinical features and family history could not offer any information of the causal underlying gene, which means complete genetic testing of all the pathogenic genes is necessary. Next-generation sequencing (NGS) offers convenience in efficiently and simultaneously analyzing multiple genes.

In this study, we performed a gene analysis of three sporadic dRTA cases diagnosed at our center by NGS. The clinical data and gene analysis of these patients are summarized and reported.

## Methods

### Patients

Three Chinese patients participated in this study. The patients’ clinical features and laboratory findings were collected. The diagnosis of primary dRTA was based on the combination of an inability to acidify urine (pH >5.5) in the setting of a normal anion gap, spontaneous metabolic acidosis, hypokalemia, and no evidence of secondary causes of dRTA. The follow-up period ranged from 1 to 4 years, and the follow-up interval was three months.

The study protocol was approved by the Ethics Committee of the Children’s Hospital of Fudan University. Informed consent for the blood collection and genetic analysis was obtained from the parents.

### Gene analysis

Peripheral blood samples were collected from the dRTA patients and their parents into EDTA tubes. The genomic DNA was extracted from 2 mL of each sample using a QIAamp Blood DNA Mini Kit (Qiagen) according to the manufacturer's instructions. Next-generation sequencing was performed by the Joy Orient Translational Medicine Research Center.

### Next-generation sequencing (NGS)

The genomic DNA samples were sheared by sonication. The sheared genomic DNA was then hybridized using a NimbleGen 2.0 probe sequence capture array obtained from Roche, (http://www.nimblegen.com/products/seqcap/ez/v2/index.html) to enrich the exonic DNA (Joy Orient, China). The library enrichment was first tested by qPCR, followed by an assessment of the size distribution and concentration using an Agilent Bioanalyzer 2100. The samples were then sequenced on an Illumina HiSeq2500 platform. Two parallel reactions were performed for each sample.

### Data filtering, mapping and variant detection

The exon-enriched DNA was sequenced using the Illumina HiSeq2500 platform following the manufacturer’s instructions (Illumina). The raw image files were processed using the BclToFastq (Illumina) for base calling and generating the raw data. The low-quality variations were filtered using the criterion of quality score ≥20 (Q20). The sequencing reads were aligned to the NCBI human reference genome (hg19) using BWA. Samtools and Pindel were used to analyze the SNPs and indels in the sequence.

### Data analysis

- Synonymous changes and SNPs (single nucleotide polymorphisms) with MAF (minor allele frequency) greater than 5% were removed (http://www.ncbi.nlm.nih.gov/projects/SNP).
- Nonsynonymous changes were filtered using SIFT software (http://sift.jcvi.org).
- The functions of the mutated genes and their relationship to the disease were analyzed.
- Sanger sequencing was performed in the analysis to confirm the mutations.
   The primers used to identify the mutations are presented in Table 1. The sequences were analyzed and compared to the reference sequences of each gene (ATP6V1B1: NM_001692, ATP6V0A4: NM_020632, and SLC4A1: NM_000342).

**Table 1.** The primers used for Sanger sequence.

Gene (exon)	Forward primer	Reverse primer
SLC4A1 (E14)	GATGATGGACGGATGAATGGATGGATAA	GCTGAGGAGTTGGACACCTTGAAG
ATP6V1B1 (E5)	TGGTGGTGTGGAGGGTAGACA	GGTTCAGTGGAAGATTTGGGGATA
ATP6V1B1 (E9)	CTCTAAACACCTGGCTACACCTC	AGACCAAGCCCTGGAACTCAT
ATP6V0A4 (E14)	CATGATAACAAATACCAGCCTAGGAC	CCCCAACCATGAAAACAGTCAC
ATP6V0A4 (E21)	CCCCTGAAACTACGTATAAGATGTTG	AGGTATGTAAGCTGCTAAAGTCAC
- The prediction of the biological function of the proteins of the reported missense mutations was performed using SIFT (http://sift.jcvi.org/), PolyPhen-2 (http://genetics.bwh.harvard.edu/pph2/), and Mutation Taster (http://www.mutationtaster.org/).

## Results

### Clinical findings

We studied three patients (one girl and two boys) from independent nonconsanguineous families who presented with the clinical features of dRTA. The clinical features are presented in Table 2. Primary dRTA was clinically diagnosed based on the clinical features (i.e., growth retardation, dehydration, and vomiting) and laboratory findings (i.e., non-gap severe metabolic acidosis, high urinary pH, hypokalemia, and nephrocalcinosis) after excluding the secondary causes of dRTA. Two patients were diagnosed at 3 months of age, and 1 patient was diagnosed at 3 years of age. The initial manifestations were either acute, with dehydration and vomiting, or failure to thrive and/or weakness. The physical examination revealed growth retardation, and both the patients’ weight and height were below the 3rd–25th percentile. Rickets was noted in Patient-3. An audiometric evaluation revealed normal hearing at diagnosis in all patients.

**Table 2.** Clinical features and laboratory tests of patients.

	Patient-1	Patient-2	Patient-3	Normal value
Age at Diagnosis	3 months	3 months	3 years	
Gender	F	M	M	
Initial manifestations	Vomiting and dehydration	Failure to thrive	Short stature and weakness	
Height/percentile（cm/th）	58/p25	54/p3	90/p3	
Weight/percentile（kg/th）	5.6/p25	4/p3	13/p10	
Blood PH	7.26	7.29	7.26	7.35–7.45
HCO_3_^−^ (mmol/L)	15.6	14.9	11.4	22–30
K^+^ (mmol/L)	2.6	3	1.9	3.5–5.5
Urinary PH	7.5	7.5	8	＜5.5
Nephrocalcinosis	Yes	Yes	Yes	
SNHL	No	No	No	

**Figure 1.**
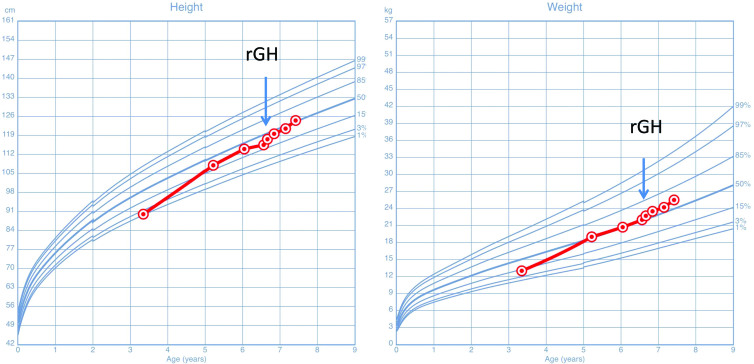
Growth condition before and after rGH therapy in patient-3. Source: Child Growth Standards (0–5 years), 2006, World Health Organization. Growth Reference (5–19 years), 2007, World Health Organization.

Acid-base homeostasis is critical for normal growth and development and the maintenance of normal cellular function. Alkali therapy is the conventional therapy used to maintain systemic homeostasis. For each patient, treatment with potassium citrate was initiated at diagnosis, and the dose of citrate was regulated based on the blood pH and biochemical findings. During the follow-up, two patients (except for Patient-3) achieved the average height with alkali therapy by the final follow-up (1 year). After three years of potassium citrate treatment, Patient-3 failed to exhibit the normal growth (<p25th). His bone age was delayed by 3 years, and the insulin stimulation tests revealed a peak serum GH concentration of 10 μg/l. Due to the poor growth and parental expectations, we initiated recombinant human growth hormone (rhGH) therapy in patient-3 at 6 years of age. The average dose of rhGH was 0.05 mg/kg daily. After 10 months of rhGH therapy, he grew at an average rate of 9.6 cm per year, which was considerable increased following the initiation of the growth hormone therapy (3 cm/yr), and he achieved the average height for his age and gender (Figure 1).

### Genetic analysis

The dRTA diagnosis of these patients was further confirmed by molecular analyses, which are important for achieving the precise diagnosis, guiding clinical therapy and prognosis, and genetic consultations. Five mutations in the dRTA genes (i.e., SLC4A1, ATP6V1B1, and ATP6VOA4) were identified in our study.

**Figure 2.**
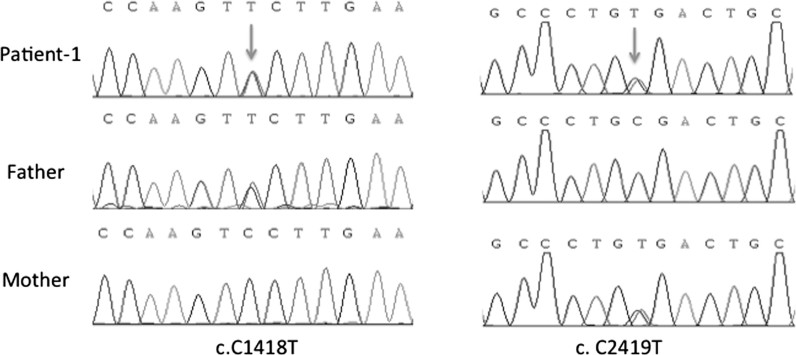
Results of sequencing for the c.1418C > T and c.2419C > T mutations in ATP6V0A4 in family-1.

Patient-1 presented with a novel compound heterozygous mutation in the ATP6VOA4 gene consisting of a mutation in exon 14 with the single nucleotide change c.1418C > T, which caused a substitution of serine acid with phenylalanine at position 473 (p.S473F), and the nonsense mutation c.2419C > T (p.R807X,34) in exon 21, which resulted in the generation of a premature stop codon. These two mutations were predicted to be damaging by SIFT, PolyPhen-2, and Mutation Taster. The patient and her parents underwent Sanger sequencing using primers designed for exons 14 and 21 of the ATP6V0A4 gene. The gene analysis confirmed these mutations and revealed that the patient inherited the former mutated allele from her father and the latter from her mother (Figure 2).

**Figure 3.**
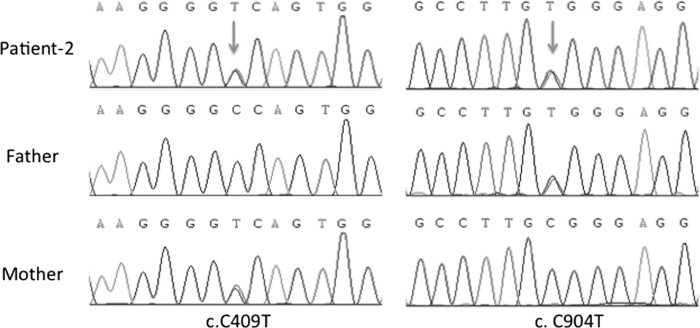
Results of sequencing for the c.409C > T and c.904C > T mutations in ATP6V1B1 gene in family-2.

Patient-2 exhibited the following compound heterozygous mutations in the ATP6V1B1 gene: two heterozygous missense mutations of c.409C > T (p. P137 S) in exon 5 and c.904C > T (p. R302 W) in exon 9, which caused the change of a cytosine to a thymine at positions 409 and 904 of the ATP6V1B1 gene coding sequence. Although the c.409C > T (p. P137 S) mutation was predicted to be benign by Polyphen-2 (score:0.38), the bioinformatics analysis using SIFT and Mutation Taster confirmed the deleterious effect of this variation (SIFT: score: 0.01, median: 3.02, and Mutation Taster revealed a *p* value of 0.999). Furthermore, at this position, the nucleotide and amino acid are highly conserved (phyloP score: 4.766, PhastCons score: 0.998). The c.904C > T (p. R302 W) mutation was predicted to be disease causing by the three different prediction tools (SIFT: score: 0.00, median: 3.02, Polyphen-2: score: 0.998, and Mutation Taster revealed a *p* value of 1). We tested the presence of these two variants in his unaffected parents and identified a c.409C > T mutation in his mother and a c.904C > T mutation in his father (Figure 3).

**Figure 4.**
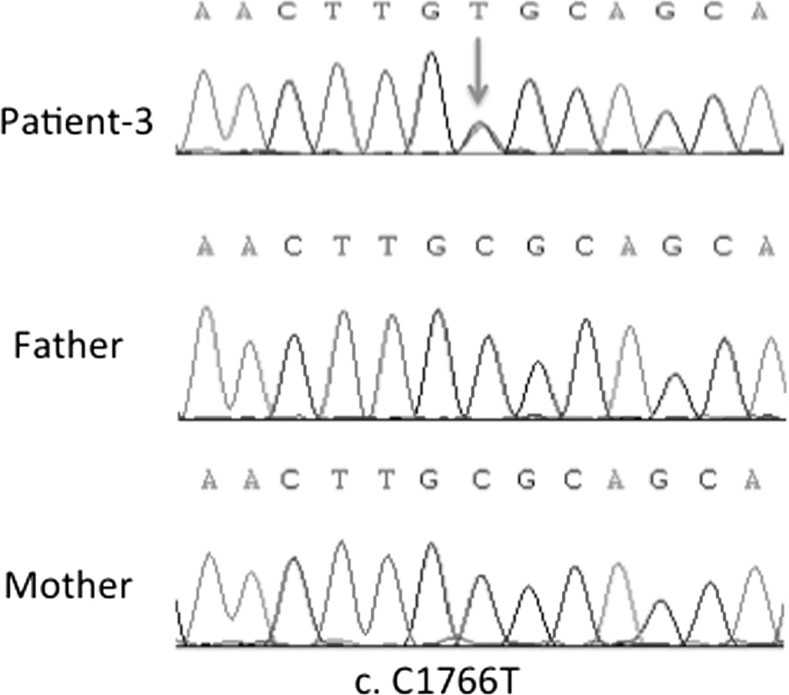
DNA sequencing profile of the c.1766C > T mutation in SLC4A1 gene in family-3.

The missense mutation in exon 14 (c.1766C > T; p. R589H) of the SLC4A1 gene was identified in Patient-3 as a heterozygous mutation. The Sanger sequencing confirmed the identified mutation in SLC4A1. The mutation identified in our series (patient-3) has been previously published [16]. The patient’s parents harbored no mutation at this position, indicating that the mutation occurred *de novo* in our patient (Figure 4).

## Discussion

dRTA results from impaired urinary acidification, leading to a series of clinical features, including inappropriate alkaline urine, metabolic acidosis with a normal anion gap and growth retardation. Both autosomal dominant and autosomal recessive forms of primary dRTA have been described. Dominant dRTA has been attributed to SLC4A1 gene defect in 1997 by Bruce [16]. In the following two years, Karet et al. [17] and Smith et al. [18] demonstrated that the AR form of dRTA was associated with mutations in the ATP6V1B1 and ATP6V0A4 genes in patients with early or absent/late sensorineural hearing loss, respectively. To date, 30 mutations in SLC4A1, 38 mutations in ATP6V1B1, and 57 mutations in ATP6V0A4, including nonsense, missense, frameshift, and splice site mutations, have been identified as causes of dRTA. All mutations are predicted to disrupt the processing and trafficking of the AE1 Cl^−^/HCO_3_^−^ exchanger or abrogate the production of the normal H+-ATPase proton pump [19–22].

In this study, 5 loss-of-function mutations were identified in our patients. Patient-1 carried a compound heterozygous mutation (p. S473F and p. R807X, 34) in the ATP6V0A4 gene, and Patient-2 harbored a compound heterozygous mutation (p. P137 S and p. R302 W) in the ATP6V1B1 gene. The deleterious predictions obtained by the bioinformatics software and the consistent autosomal recessive inheritance of the mutations from the unaffected parents indicate that the ATP6VOA4 and ATP6V1B1 compound heterozygous mutations are the genetic causes of dRTA in Patient-1 and Patient-2, respectively. Unlike the reported homozygous mutations of dRTA cases from high rate of consanguinity, our patient shows compound heterozygous mutations, which was due to the ban of consanguineous marriage and the ethics.

Defects in ATP6V1B1 are believed to cause dRTA with SNHL, whereas defects in ATP6V0A4 are responsible for cases without or late onset SNHL [3,17]. Our patients harboring the ATP6V1B1/ATP6VOA4 mutation exhibited normal hearing at diagnosis. Since our patients are young, long-term follow-up of these two patients is necessary to confirm the development and severity of hearing loss due to these novel mutations.

Patient-3, who harbors a *de novo* mutation (R5889H) in the SLC4A1 hotpot position [16], was diagnosed with dRTA at the age of 3, which is considerably older than the other patients who carried the ATP6V0A4 or ATP6V1B1 gene mutation. This result is consistent with previous records reporting that patients with recessive dRTA are severely affected and often diagnosed at a young age, whereas dominant dRTA is diagnosed at an older age, suggesting that cases with late clinical onset dRTA harbor SLC4A1 gene mutations.

Remarkably, the p. P137S and p. R302W mutations in ATP6V1B1 and p. S473F and p. R807X mutations in ATP6V0A4 have not been previously reported. These findings expand the spectrum of mutations in the SLC4A1, ATP6V1B1, and ATP6V0A4 genes associated with primary dRTA and provide insight into possible phenotype–genotype correlations.

Growth impairment is a major problem in children with renal tubular acidosis worthy of our concern. Acid-base homeostasis is critical for normal growth and development. Commonly, alkali supplementation can correct the systemic metabolic defects, restore a normal acid-base balance, and improve growth [23–25]. Although early treatment corrects the biochemical abnormalities and helps foster normal growth, the final height remains compromised in dRTA patients (the mean height SDS was −1.1 for patients diagnosed during infancy and −2 for those diagnosed later) [26,27]. Consistent with previous findings in which the catch-up growth was limited to the first 2 years of therapy in patients treated before 2 years of age, the first two patients, who were diagnosed at 3 months, exhibited significant growth, whereas Patient-3, who was diagnosed at 3 years of age, failed to exhibit catch-up growth during the three-year follow-up. In Patient-3, an endocrine evaluation revealed a short stature with unimpaired growth hormone secretion, and combined conventional alkali supplementation with rGH therapies improved his growth rate. Recombinant GH therapy in patients with a short stature with GHD and other disorders that do not fit the definition of classic GH deficiency, such as intrauterine growth restriction, healthy children with a short stature or chronic renal disease (CKD), has been reported to have a beneficial effect on growth with no adverse effects on renal function [28–30]. To the best of our knowledge, this is the first case in the literature describing the use of growth hormone therapy in a patient with poor growth associated with genetically diagnosed dRTA. Although the results were associated with a single case, our results inferred the beneficial effect of rGH therapy on patients with inherited distal renal tubular disease. Further studies involving more patients are needed to confirm this result.

## Conclusions

In our study, we confirm the effect of early treatment in improving growth for dRTA patient and suggest that rGH therapy may have a beneficial effect on growth in dRTA patients who were diagnosed late and have a persistent growth delay. Overall, five different mutations were identified, and four mutations, i.e., the p. P137S and p. R302W mutations in ATP6V1B1 and p. S473F and p. R807X in ATP6V0A4, were novel disease-causing mutations. The identification of these mutations extended the spectrum of gene mutations associated with primary dRTA.

## Disclosure statement

No potential conflict of interest was reported by the authors.

## Abbreviations

dRTA: distal renal tubular acidosis
rGH: recombined growth hormone
NGS: next-generation sequencing.

## References

[1] Rodriguez-Soriano J, Vallo A Renal tubular acidosis. Pediatr Nephrol. 1990;4:268–275. 2205272 10.1007/BF00857675

[2] Karet FE Inherited distal renal tubular acidosis. J Am Soc Nephrol. 2002;13:2178–2184. 12138152 10.1097/01.asn.0000023433.08833.88

[3] Stover EH, Borthwick KJ, Bavalia C, et al. Novel ATP6V1B1 and ATP6V0A4 mutations in autosomal recessive distal renal tubular acidosis with new evidence for hearing loss. J Med Genet. 2002;39:796–803. 12414817 10.1136/jmg.39.11.796 PMC1735017

[4] Ruf R, Rensing C, Topaloglu R, et al. Confirmation of the ATP6B1 gene as responsible for distal renal tubular acidosis. Pediatr Nephrol. 2003;18:105–109. 12579397 10.1007/s00467-002-1018-8

[5] Feldman M, Prikis M, Athanasiou Y, et al. Molecular investigation and long-term clinical progress in Greek Cypriot families with recessive distal renal tubular acidosis and sensorineural deafness due to mutations in the ATP6V1B1 gene. Clin Genet. 2006;69:135–144. 16433694 10.1111/j.1399-0004.2006.00559.x

[6] Gil H, Santos F, Garcia E, et al. Distal RTA with nerve deafness: clinical spectrum and mutational analysis in five children. Pediatr Nephrol. 2007;22:825–828. 17216496 10.1007/s00467-006-0417-7

[7] Khositseth S, Sirikanerat A, Wongbenjarat K, et al. Distal renal tubular acidosis associated with anion exchanger 1 mutations in children in Thailand. Am J Kidney Dis. 2007;49:841–850.e841. 17533027 10.1053/j.ajkd.2007.03.002

[8] Anacleto FE, Bruce LJ, Clayton P, et al. Distal renal tubular acidosis in Filipino children, caused by mutations of the anion-exchanger SLC4A1 (AE1, Band 3) gene. Nephron Physiol. 2010;114:p19–p24. 20068363 10.1159/000274484

[9] Elhayek D, Perez de Nanclares G, Chouchane S, et al. Molecular diagnosis of distal renal tubular acidosis in Tunisian patients: proposed algorithm for Northern Africa populations for the ATP6V1B1, ATP6V0A4 and SCL4A1 genes. BMC Med Genet. 2013;14:119. 24252324 10.1186/1471-2350-14-119 PMC4225572

[10] Hooman N, Otukesh H, Fazilaty H, et al. A novel mutation pattern of kidney anion exchanger 1 gene in patients with distal renal tubular acidosis in Iran. Iran J Kidney Dis. 2015;9:230–238. 25957428

[11] Gomez J, Gil-Pena H, Santos F, et al. Primary distal renal tubular acidosis: novel findings in patients studied by next-generation sequencing. Pediatr Res. 2016;79:496–501. 26571219 10.1038/pr.2015.243

[12] Besouw MTP, Bienias M, Walsh P, et al. Clinical and molecular aspects of distal renal tubular acidosis in children. Pediatr Nephrol. 2017;32:987–996. 28188436 10.1007/s00467-016-3573-4

[13] Palazzo V, Provenzano A, Becherucci F, et al. The genetic and clinical spectrum of a large cohort of patients with distal renal tubular acidosis. Kidney Int. 2017;91:1243–1255. 28233610 10.1016/j.kint.2016.12.017

[14] Gao Y, Xu Y, Li Q, et al. Mutation analysis and audiologic assessment in six Chinese children with primary distal renal tubular acidosis. Ren Fail. 2014;36:1226–1232. 24975934 10.3109/0886022X.2014.930332

[15] Du J, Pang QQ, Jiang Y, et al. [Clinical features of hereditary distal renal tubular acidosis and SLC4A1 gene mutation]. Zhongguo dang dai er ke za zhi = Chin J Contemp Pediatr. 2017;19:381–384. 10.7499/j.issn.1008-8830.2017.04.003 PMC7389662 28407820

[16] Bruce LJ, Cope DL, Jones GK, et al. Familial distal renal tubular acidosis is associated with mutations in the red cell anion exchanger (Band 3, AE1) gene. J Clin Investig. 1997;100:1693–1707. 9312167 10.1172/JCI119694 PMC508352

[17] Karet FE, Finberg KE, Nelson RD, et al. Mutations in the gene encoding B1 subunit of H+-ATPase cause renal tubular acidosis with sensorineural deafness. Nat Genet. 1999;21:84–90. 9916796 10.1038/5022

[18] Smith AN, Skaug J, Choate KA, et al. Mutations in ATP6N1B, encoding a new kidney vacuolar proton pump 116-kD subunit, cause recessive distal renal tubular acidosis with preserved hearing. Nat Genet. 2000;26:71–75. 10973252 10.1038/79208

[19] http://www.HGMD.CF.AC.UK/.

[20] Fry AC, Su Y, Yiu V, et al. Mutation conferring apical-targeting motif on AE1 exchanger causes autosomal dominant distal RTA. J Am Soc Nephrol. 2012;23:1238–1249. 22518001 10.1681/ASN.2012020112 PMC3380654

[21] Bruce LJ, Unwin RJ, Wrong O, et al. The association between familial distal renal tubular acidosis and mutations in the red cell anion exchanger (band 3, AE1) gene. Biochem Cell Biol. 1998;76:723–728. 10353704 10.1139/bcb-76-5-723

[22] Cordat E, Kittanakom S, Yenchitsomanus PT, et al. Dominant and recessive distal renal tubular acidosis mutations of kidney anion exchanger 1 induce distinct trafficking defects in MDCK cells. Traffic. 2006;7:117–128. 16420521 10.1111/j.1600-0854.2005.00366.x

[23] Santos F, Chan JC Renal tubular acidosis in children. Diagnosis, treatment and prognosis. Am J Nephrol. 1986;6:289–295. 3777038 10.1159/000167177

[24] Pirojsakul K, Tangnararatchakit K, Tapaneya-Olarn W Clinical outcome of children with primary distal renal tubular acidosis. J Med Assoc Thai. 2011;94:1205–1211. 22145505

[25] Cachat F, Froidevaux V, Guignard JP [Renal tubular acidosis in children]. Pediatrie 1993;48:313–326. 8397383

[26] Caldas A, Broyer M, Dechaux M, et al. Primary distal tubular acidosis in childhood: clinical study and long-term follow-up of 28 patients. J Pediatr. 1992;121:233–241. 1640289 10.1016/s0022-3476(05)81194-1

[27] Bajpai A, Bagga A, Hari P, et al. Long-term outcome in children with primary distal renal tubular acidosis. Indian Pediatr. 2005;42:321–328. 15876593

[28] Berard E, Andre JL, Guest G, et al. Long-term results of rhGH treatment in children with renal failure: experience of the French Society of Pediatric Nephrology. Pediatr Nephrol. 2008;23:2031–2038. 18584215 10.1007/s00467-008-0849-3

[29] Haffner D, Schaefer F, Nissel R, et al. Effect of growth hormone treatment on the adult height of children with chronic renal failure. German Study Group for Growth Hormone Treatment in Chronic Renal Failure. N Engl J Med. 2000;343:923–930. 11006368 10.1056/NEJM200009283431304

[30] Vimalachandra D, Hodson EM, Willis NS, et al. Growth hormone for children with chronic kidney disease. Cochrane Database Syst Rev. 2012. doi:10.1002/14651858.CD003264.pub3 PMC6599873 22336787

